# Supplementary material for: Dynamic stiffening of the flagellar hook
Source: Nat Commun. 2022 May 25;13:2925. doi: 10.1038/s41467-022-30295-7 (PMC9133114; doi:10.1038/s41467-022-30295-7)
Supplement: Supplementary file 1 — Supplementary Information [file 41467_2022_30295_MOESM1_ESM.pdf]

Dynamic stiffening of the flagellar hook

Supplementary Information

Ashley L. Nord, Anaïs Biquet-Bisquert, Manouk Abkarian, Théo Pigaglio, Farida Seduk, Axel Magalon, Francesco Pedaci

Contents

1 Supplementary Figures **2**

2 Supplementary Methods **5**

2.1 Drag coefficients 5

2.1.1 Plane (r,z) 6

2.1.2 Plane parallel to (x,y) 7

2.2 Analysis workflow 8

2.3 Corrections of the bead trajectory 11

Supplementary Notes **12**

1. Simulating a particle in a harmonic potential in presence of a drag gradient . 12

2. Bend-twist coupling, persistence length, and Young's modulus 14

3. Effect of torque fluctuations 16

4. Effect of centrifugal force 16

5. Comparison of hook bending stiffening under opposite twists 18

5.1 cheRB mutant preparation 19

1 Supplementary Figures

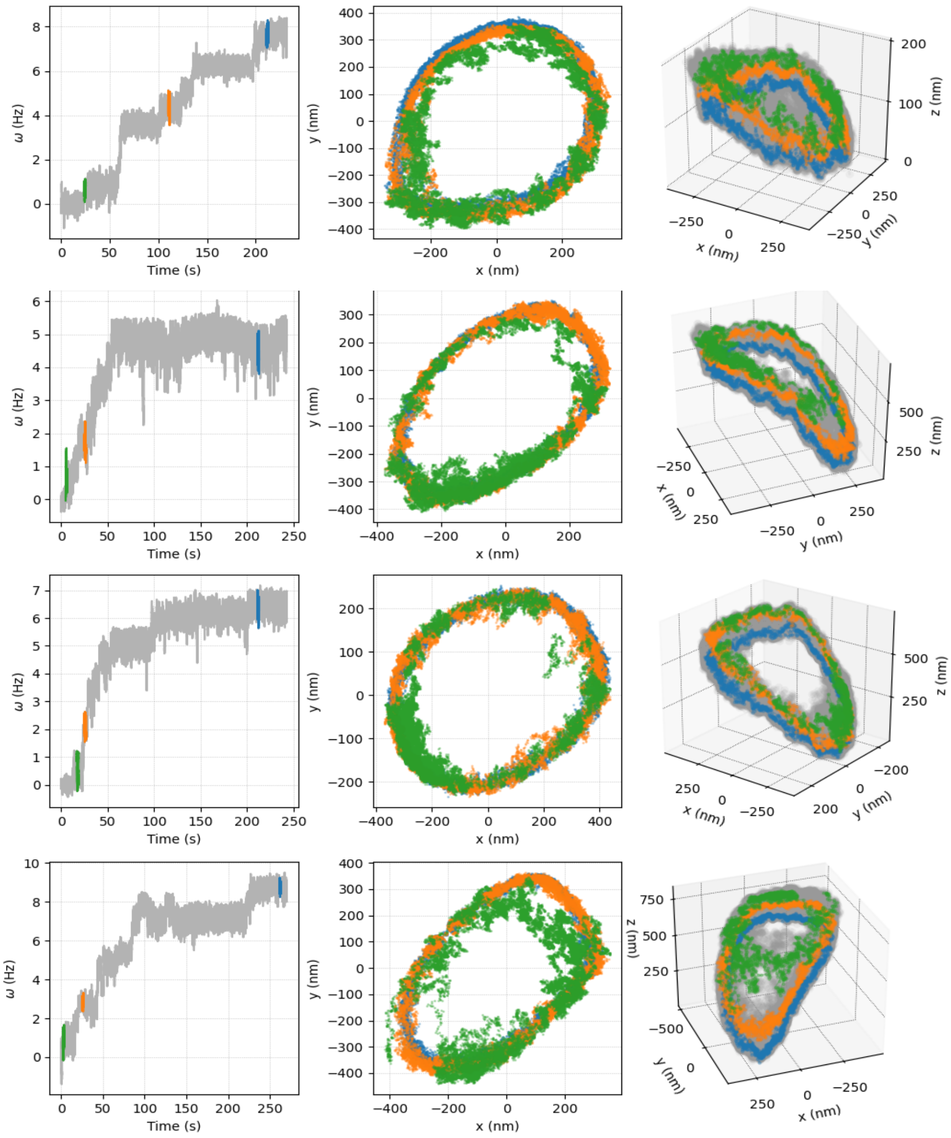

Supplementary Figure 1: Examples of 3D trajectories of beads (R_b = 1000 nm). Each line corresponds to a different bead. The left column shows the angular speed $\omega(t)$, where the resurrection of the motor is evident. Three particular time-windows are highlighted in green, orange and blue. The middle column shows the xy projection of the trajectory, where the colors correspond to the highlighted time-windows. At early times (green) the rotation of the bead is more erratic. The right column shows the trajectory in 3 dimensions.

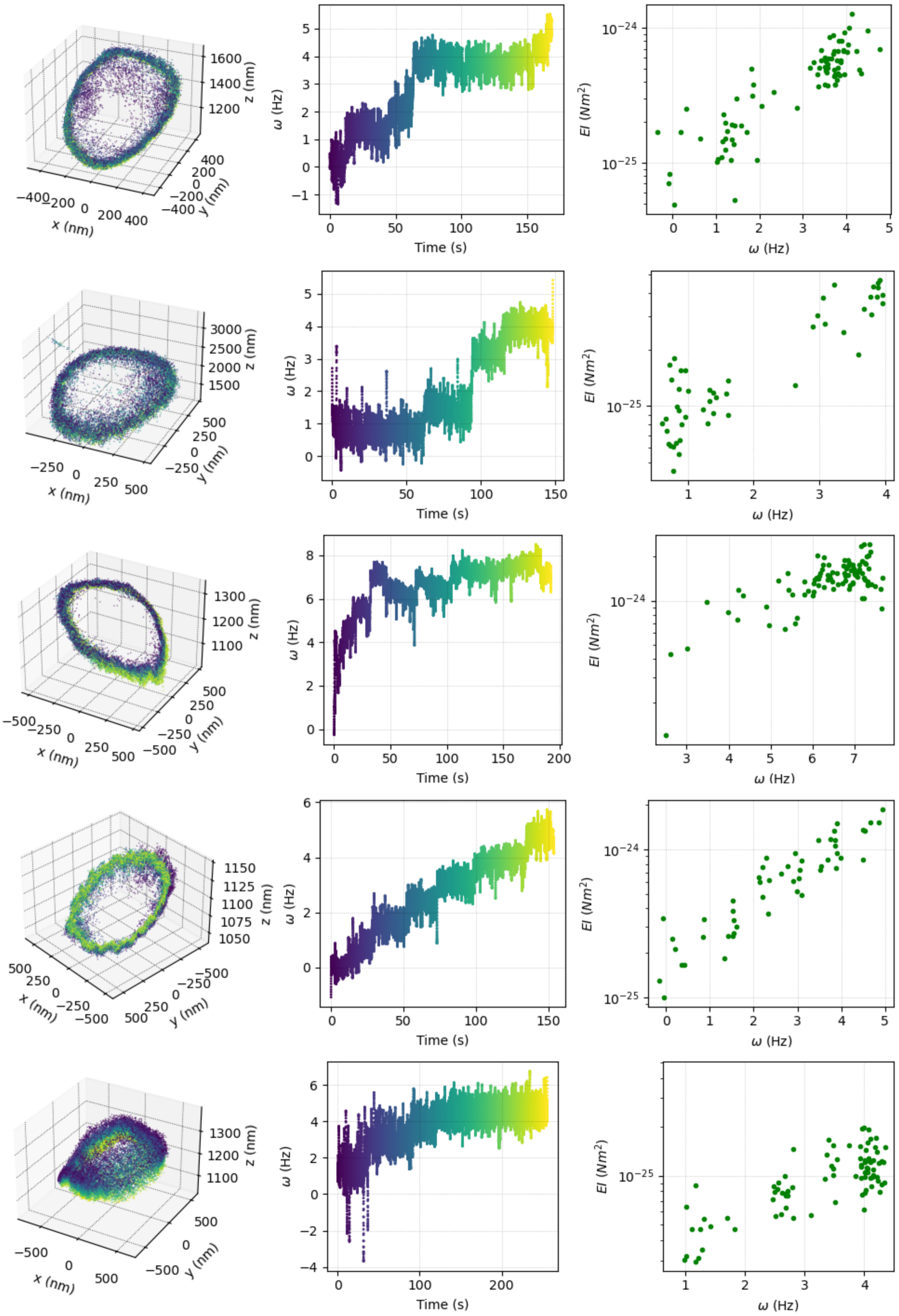

Supplementary Figure 2: Examples of the result of the analysis leading to the value of the bending stiffness EI taking into account the full 3D trajectory of the bead. Each line corresponds to a different motor bound to a $R_b = 1000$ nm bead. In each row, the left panel shows the recorded 3D trajectory of the bead center. The middle panel shows the angular speed $\omega(t)$ during resurrection. In the left and middle panels the color from dark to yellow indicates time. The right panel shows the resulting bending stiffness $EI(\omega)$ calculated considering the 3D trajectory, and defining θ and L from x, y, z (For more details, see the "case xyz" in the analysis workflow of sec.2.2). The second to last line shows one x, y, z trajectory with an inverted convex geometry. Like the others, the bead rotates in the CCW direction, and EI increases with speed. This is a rare case, for which we do not have a clear explanation.

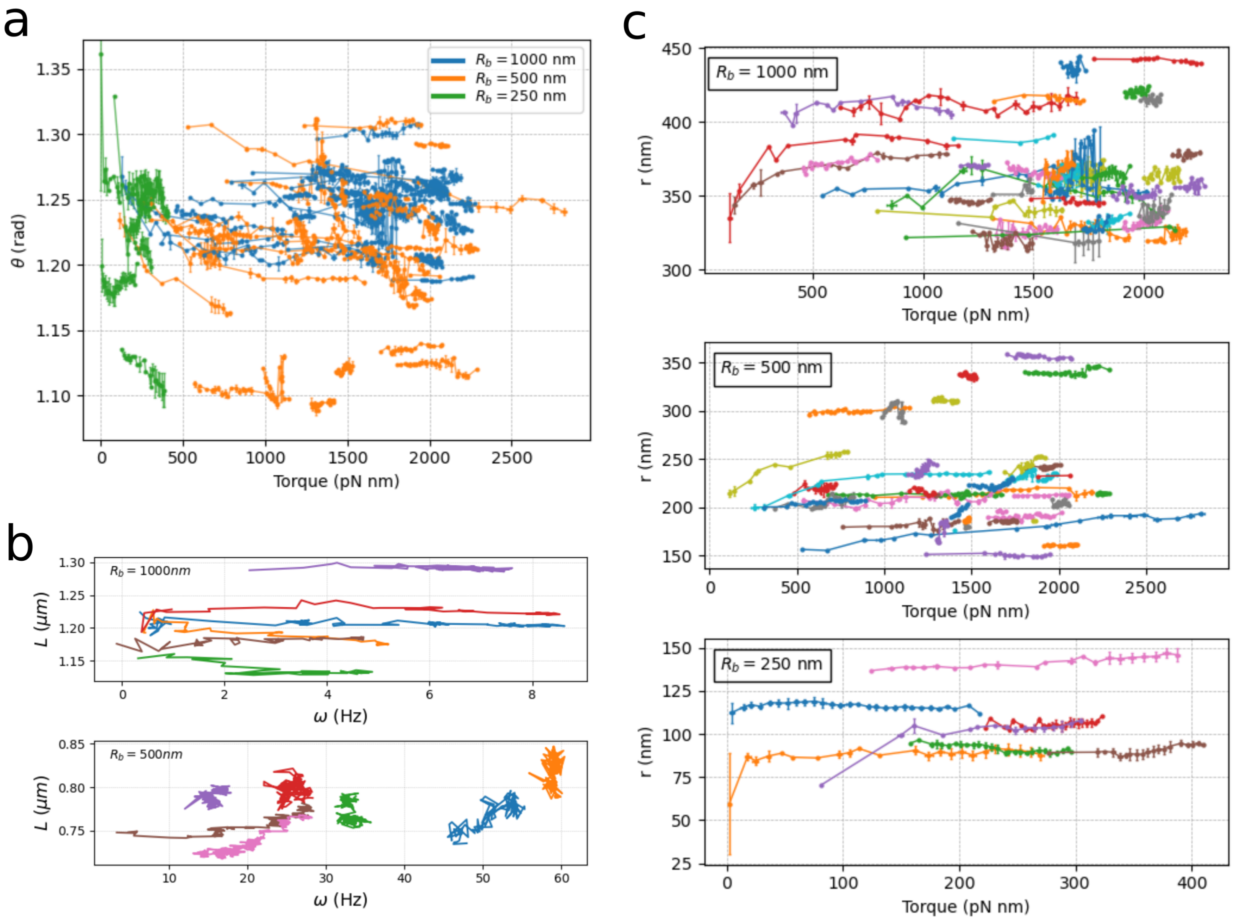

Supplementary Figure 3: Geometrical analysis of all the traces recorded (see Fig.4 for the geometrical definitions). Based on these observations we formulate the simplifying hypothesis that the bead rotates on hemispherical dome, approaching the membrane as the rotation speed increases. a) The angle θ, averaged in each time-window of each trace, is shown as a function of the measured motor torque. The three colors indicate the three bead sizes employed, as indicated. Generally, and especially starting from low torque, as the motor accelerates during resurrection, the bead moves down towards the membrane, reflected by a decrease in θ. b) Values of L (distance between bead center and hook origin on the membrane, averaged on short 0.5-1 s time-windows along the traces) extracted from x, y, z bead trajectories, as a function of angular speed, for beads with $R_b = 1000, 500$ nm. For a given bead in a 3D trajectory, L remains reasonably constant. c) For the same data shown in a) we show the radius r of the x, y trajectory, averaged in each time-window, as a function of motor torque. The movement of the bead towards the membrane is reflected here by an increase in r for a particular trajectory. The three loads are split in the three panels, as indicated. In a) and c), the measurements, each on a different cell, consist of 36, 35, and 7 traces for motors at steady state and 6, 13, and 6 resurrection traces for beads of radius $R_b = 1000, 500, 250$ nm, respectively, and are presented as mean values ± SD in each time-window.

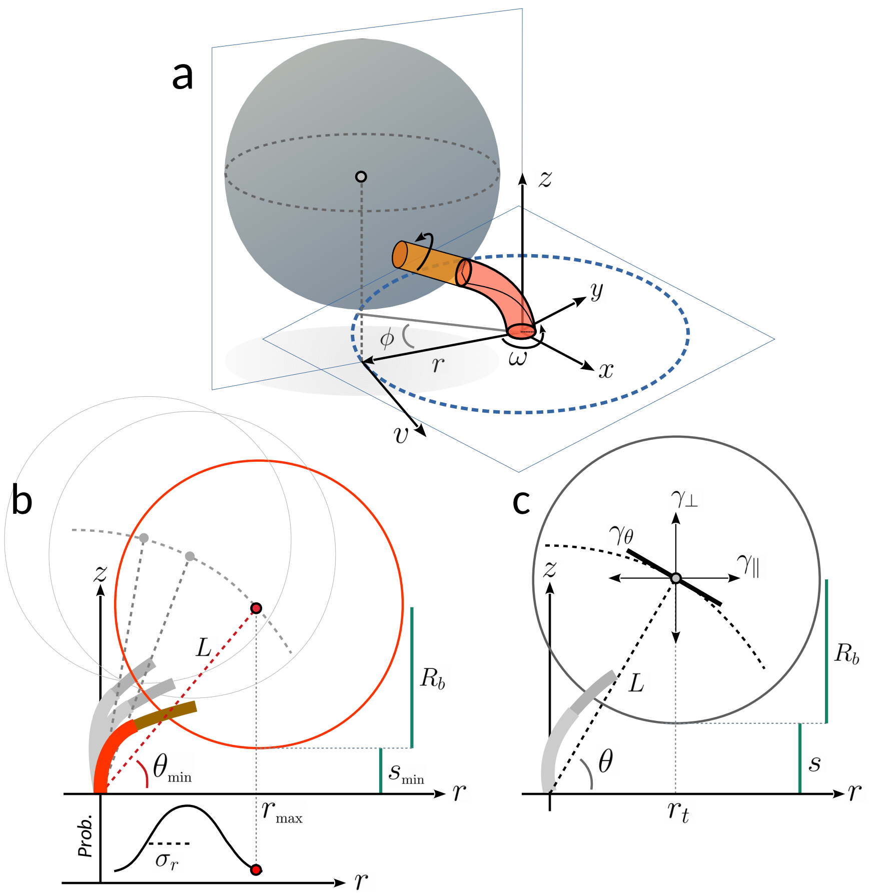

Supplementary Figure 4: Microscopic geometrical model. a) 3D representation of the bead (not to scale) tethered to the flagellar stub, composed by the hook (red, length of 60 nm) and filament (FliCst, orange, of 20 nm diameter [11]). The angle ϕ describes the motion of the center of the bead along the circular trajectory of radius r. The linear speed of the bead is $v = \omega r$, where the angular velocity is $\omega = d\phi/dt$. b) Projection on the (z, r) plane, where the center of the bead is assumed to move on an arc of radius L, described by the angle θ. The probability distribution of r (approximated by a Gaussian of width σ_r) is shown in the graph below. The minimum value of θ visited in an entire measurement ($\theta_{\min}$) corresponds to the maximum visited value of the radius $r_{\max}$, and to the minimum distance $s_{\min}$ between the bead surface and the membrane. c) Same as in b) for a generic position (r_t, θ) at time t. The drag coefficient on the plane (z, r) is composed by a parallel ($\gamma_\parallel$) and perpendicular ($\gamma_\perp$) component with respect to the membrane, which are projected (γ_θ) on the direction tangent to the arc of radius L. The distance between the bead surface and the membrane is $s(t) \geq s_{\min}$. The radius of the bead is R_b.

2 Supplementary Methods

2.1 Drag coefficients

Here we aim at writing the corrections to the drag coefficients due to the proximity with surfaces. In this attempt, we simplify the geometry assuming an infinite plane hard wall, with the hook exiting perpendicularly from it. The actual system is clearly more complicated: the elongated cell has a length of a couple microns and a diameter of ~ 0.5 μm; therefore, while along the axial direction it could be considered flat (when using micron-size beads), in the perpendicular direction, the surface curvature is comparable with the bead size. Moreover, the actual position of the motor on the cell surface, and consequently the presence and relative position of the flat glass surface, are also relevant, but have been neglected for simplicity and because we do not have direct access to them.

2.1.1 Plane (r,z)

We aim at writing the expression of the bead angular drag coefficient γ_θ in the direction tangent to the arc trajectory of radius L, in the plane (r, z), described by the angle θ (Fig.4). We consider the movement of the center of the bead along the arc, where the linear tangential speed is $v_{tg} = \dot{\theta}L$, and where the force acting on the bead is $F_{tg} = v_{tg}\gamma_{tg}$, with γ_{tg} the linear drag coefficient in the tangential direction. The associated torque can then be written as $\tau_\theta = \gamma_\theta\dot{\theta} = F_{tg}L = v_{tg}\gamma_{tg}L = \gamma_{tg}L^2\dot{\theta}$. The linear drag γ_{tg} is composed by the parallel and perpendicular components, as[1] $\gamma_{tg} = \gamma_{\|}\sin^2\theta + \gamma_\perp\cos^2\theta$. Therefore, the angular drag γ_θ can be written as

$$\gamma_\theta = \gamma_\theta(\theta, s, R_b) = L^2(\gamma_{\|}\sin^2\theta + \gamma_\perp\cos^2\theta). \tag{1}$$

The components $\gamma_{\|}$ and $\gamma_\perp$ can be written following the treatment developed by Faxen or Brenner, given below. In Fig. 5 we show the value of these components as a function of the gap s between the wall and the bead (of radius $R_b = 500$ nm). In our analysis we calculate γ_θ from both theories (Faxen and Brenner), and for every θ (or s) we take the maximum of the two.

Faxen expressions

Following the theory by Faxen [8, 12], the drag components can be written as,

$$\gamma_{\|,F} = \frac{\gamma_o}{1 - \frac{9}{16}\frac{R_b}{d} + \frac{1}{8}(\frac{R_b}{d})^3} \tag{2}$$

$$\gamma_{\perp,F} = \frac{\gamma_o}{1 - \frac{9}{8}(\frac{R_b}{d}) + \frac{1}{2}(\frac{R_b}{d})^3}, \tag{3}$$

where $\gamma_o = 6\pi\eta R_b$ is the drag of a spherical particle in the bulk (far from surfaces), η is the medium viscosity, R_b is the radius of the particle, and $d = s + R_b$ is the distance between the bead center and the wall.

Brenner expressions

Following Brenner theory [6, 3], the drag components are,

$$\gamma_{\perp,B} = \gamma_o C_\perp(s, R_b) \tag{4}$$

$$\gamma_{\|,B} = \gamma_o C_{\|}(s, R_b), \tag{5}$$

where R_b is the radius of the spherical particle. $C_\perp, C_{\|}$ are correction factors for the bulk drag γ_o, functions of R_b and of the gap s between the bead surface and the membrane. Their expressions read,

$$C_{\|}(s, R_b) = \frac{8}{15}\ln\left(\frac{s}{R_b}\right) - 0.9588 \tag{6}$$

$$C_\perp(s, R_b) = \frac{4}{3}\sinh(\alpha)\sum_n \frac{n(n+1)\,C_n}{(2n-1)(2n+3)}, \tag{7}$$

[1] In the plane (x, z), the total force at a point $x = L\cos\theta$, $z = L\sin\theta$ can be written as $\vec{F} = -\gamma_{\|}\dot{x}\vec{u}_x - \gamma_\perp\dot{z}\vec{u}_z = \gamma_{\|}L\sin(\theta)\dot{\theta}\vec{u}_x - \gamma_\perp L\cos(\theta)\dot{\theta}\vec{u}_z$. The force component along θ is $F_\theta = -L(\gamma_{\|}\sin^2\theta + \gamma_\perp\cos^2\theta)\dot{\theta} = -L\gamma_{tg}\dot{\theta}$.

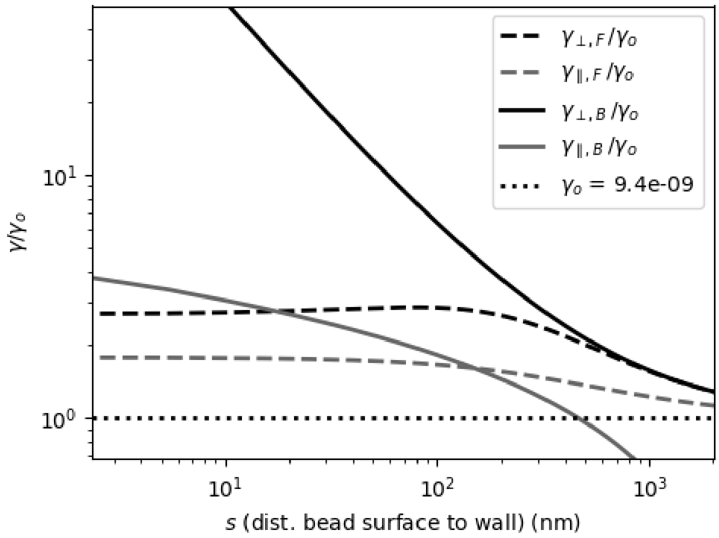

Supplementary Figure 5: Comparison between the expressions of the drag components of a bead with radius $R_b = 500$ nm from Faxen (eqs.2, 3) and Brenner theory (eq. 4, 5) as indicated in the plot legend. The bulk drag is $\gamma_o = 6\pi\eta R_b$ and its value is indicated in the legend in Ns/m.

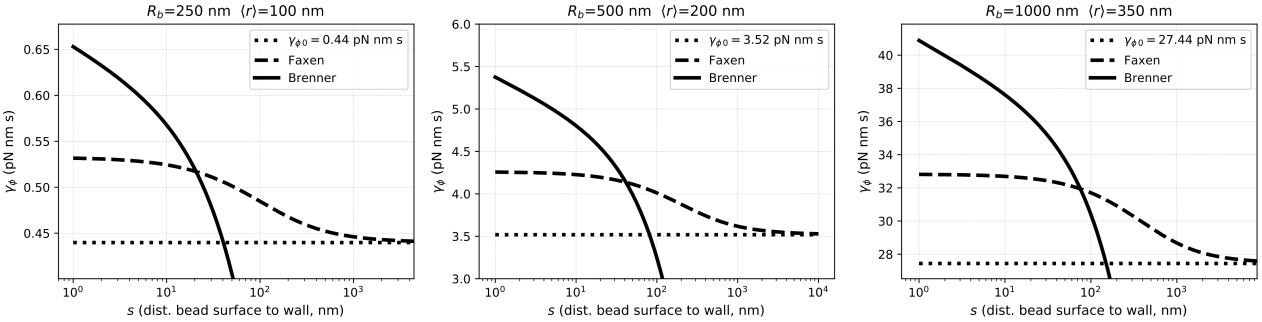

Supplementary Figure 6: Comparison between the expressions of $\gamma_{\phi,F}$ (Faxen, eq.10) and $\gamma_{\phi,B}$ (Brenner, eq.11) as a function of the gap s between the bead and the wall, for a bead of radius R_b translating and rotating on a circular trajectory (parallel to the plane x, y) of radius $\langle r \rangle$ (with the same face pointing the center), as indicated in the title of each panel. The bulk drag is defined as $\gamma_{\phi\,0} = 8\pi\eta R_b^3 + 6\pi\eta R_b \langle r \rangle^2$

where, in the expression of $C_\perp(s, R_b)$, we use $n = 1, ..., 20$ and

$$C_n = \left[\frac{2\sinh((2n+1)\alpha) + (2n+1)\sinh(2\alpha)}{4\sinh^2((n+\frac{1}{2})\alpha) - (2n+1)^2\sinh^2(\alpha)} - 1 \right] \tag{8}$$

$$\alpha = \ln\left(\frac{s+R_b}{R_b} + \sqrt{\left(\frac{s+R_b}{R_b}\right)^2 - 1} \right). \tag{9}$$

2.1.2 Plane parallel to (x,y)

For the rotation described by the angle ϕ (Fig. 4a), the drag γ_ϕ includes one component for the rotation of the bead around its axis (proportional to $8\pi\eta R_b^3$), and one component for its

rotation along a circle of radius $\langle r \rangle$ (proportional to $6\pi\eta R_b \langle r \rangle^2$). Two formalisms provide an analytical expression for the drag corrected due to proximity with a rigid wall. The Faxen expression for the corrected drag coefficient is valid for $s \geq R_b$, and can be written as [8]

$$\gamma_{\phi,F} = \frac{8\pi\eta R_b^3}{1 - \frac{1}{8}(\frac{R_b}{R_b+s})^3} + \frac{6\pi\eta R_b \langle r \rangle^2}{1 - \frac{9}{16}(\frac{R_b}{R_b+s}) + \frac{1}{8}(\frac{R_b}{R_b+s})^3} \quad . \tag{10}$$

The expression due to Brenner is valid for $s \ll R_b$ and reads

$$\gamma_{\phi,B} \;=\; 8\pi\eta R_b^3 \left(1.20205 - 3\left(\frac{\pi^2}{6} - 1\right)\frac{s}{R_b}\right) + 6\pi\eta R_b \langle r \rangle^2 \left(\frac{8}{15}\log(\frac{s}{R_b}) - 0.9588\right) \tag{11}$$

We note that the Faxen expression $\gamma_{\phi,F}$ has the advantage to remain finite for every value of s, while $\gamma_{\phi,B}$ diverges outside its range of validity (Fig. 6). For $s/R_b \to 0$, the Faxen expression $\gamma_{\phi,F}$ (outside its range) remains lower than the more accurate Brenner expression $\gamma_{\phi,B}$ by $\sim 20\%$ for the beads we employ. Therefore, despite the widespread use of $\gamma_{\phi,F}$ (e.g. in the optical trapping and BFM literature), one should be careful not to employ it for distances much smaller than the bead radius, where $\gamma_{\phi,B}$ should be preferred, in order to not under estimate the motor torque ($\tau = \gamma_\phi \omega$). In our analysis, we can extract the distance s in each time window of the traces, and for a given s we use the drag,

$$\gamma_\phi(s) = \max\left(\gamma_{\phi,F}(s), \gamma_{\phi,B}(s)\right). \tag{12}$$

2.2 Analysis workflow

We describe here in detail the workflow followed to extract all the parameters from the experimental traces. The data consist in the tracked position of the center of the bead $x(t), y(t)$ (labeled 'case xy' in the analysis below). In a smaller number of cases we also have the z position of the bead (labeled 'case xyz'). In this case, the analysis can rely directly on $z(t)$. Overall, the goal of the analysis described below is to extract the angle $\theta(t)$ in small non-overlapping time-windows $\theta_i(t)$ along the trace, which reflects the changes in time of the locally averaged bending of the hook. Non-overlapping windows form a set of independent measurements, and are beneficial to decrease the computational time. The fluctuations of $\theta_i(t)$, via its probability distribution and spectrum, together with our geometrical assumptions, provide a measurement of the hook bending stiffness EI, and its variation with motor speed, motor torque and hook twist change.

For each trace we run the following analysis, described below as a function in pseudo code termed `radial_analysis()`, which accepts as inputs the traces $x(t), y(t)$ (optionally $z(t)$), and the offset distance s_{min}. As the x, y, z bead positions are measured relatively to an arbitrary origin, the distance between the bead and the membrane is unknown. One goal of the analysis is to estimate this distance by quantifying the offset s_{min}. Once we determine s_{min}, all the geometrical variables can be determined from $x, y, (z)$. In particular:

- in each time-window i, $\theta_i(t)$ and the fit of its spectrum provides the experimental value of the drag $\gamma_{\theta,i}$
- the distance d_i between the bead center and the membrane can be fixed allowing the calculation of the theoretical drag $\gamma_{\theta,th,i}$ in the time window.

For an arbitrary choice of $s_{\rm min}$, the values of the experimental $\{\gamma_{\theta,i}\}$ and theoretical $\{\gamma_{\theta,th,i}\}$ drag, along the time windows of one trace, are different. For example, if $s_{\rm min}$ is assumed to be too large, the theoretical $\{\gamma_{\theta,th,i}\}$ will contain only the contribution of the bulk drag, while the measured $\{\gamma_{\theta,i}\}$ will be larger, due to the proximity of the bead to the membrane. For a given input $s_{\rm min}$, the function `radial_analysis(`$s_{\rm min}$`)` calculates the values of $\{\gamma_{\theta,i}\}$ and $\{\gamma_{\theta,th,i}\}$ along the input trace, and the mean square error (MSE) between them. A subsequent automatic procedure, calling `radial_analysis(`$s_{\rm min}$`)` multiple times, varies the parameter $s_{\rm min}$ in order to minimize the MSE. The value of $s_{\rm min}$ that minimizes the MSE is finally retained.

Function `radial_analysis`($x, y(, z)$ [arrays], $s_{\rm min}$ [floating point]):

1. (optional) case xyz: remove outlier points in z (which can arise when the tracker fails to converge on one frame)
2. remove drift in $x, y(, z)$ using a trace simultaneously recorded of a surface-immobilized bead located in the same field of view
3. Scale the entire x, y trajectory into a circle, fitting it to an ellipse and scaling the minor axis to become equal to the major axis
4. case xyz:
   (a) multiply z by the refraction index correction factor (0.85) [7]
   (b) (optional) correct the 1-turn periodic modulation of z (arising from a non-circular trajectory, see SI sec.2.3)
   (c) modify the value of z by shifting it vertically, $z \to z - min(z) + R_b + s_{\rm min}$, so z indicates the distance between the bead center and the cell membrane. The values of z depend now on the choice of $s_{\rm min}$
   (d) define $\theta = \arctan(z/\sqrt{x^2+y^2})$
5. given window size ($0.5 - 4$ s depending on the size of the attached particle), set the time windows from which the windowed arrays $x_i, y_i, (z_i, \theta_i)$ are defined
6. case xy:
   (a) on each window i, center the trajectory: $x_i \to x_i - \langle x_i \rangle$, and $y_i \to y_i - \langle y_i \rangle$
   (b) on each window, find the values of the radius $r_i = \sqrt{x_i^2 + y_i^2}$.
   (c) find the value of $r_{\rm max}$ (one value for the entire trace)
   (d) find the value of $L = \sqrt{(R_b + s_{\rm min})^2 + r_{\rm max}^2}$ (one value for the entire trace)
7. Main loop. On each time window i:
   (a) center the trajectory: $x_i \to x_i - \langle x_i \rangle$, and $y_i \to y_i - \langle y_i \rangle$
   (b) calculate the values of the following arrays
      - $\phi_i = \arctan(y_i/x_i)$, the tangential angle

- $\omega_i = d\phi_i/dt$, the angular speed of the bead
- $r_i = \sqrt{x_i^2 + y_i^2}$, the radius of the trajectory

(c) correct the 1-turn periodic modulation of r_i (SI sec.2.3)

(d) find the values $\theta_i(t)$ of the angle θ in the current time-window i

- case xy: $\theta_i = \arccos(r_i/L)$, where L is defined in 6d
- case xyz: θ_i windowed from θ defined in 4d

(e) calculate $PSD_{\theta_i}(f)$, the single-sided power spectral density of $\theta_i(t)$, function of frequency f

(f) fit the experimental $PSD_{\theta_i}(f)$ with the theoretical expression $PSD(f) = \frac{k_B T}{\pi^2 \gamma_\theta (f^2 + f_c^2)}$ (we use the python function `scipy.optimize.differential_evolution`), to obtain in each window i the corner frequency $f_{c,i}$ and the experimental drag $\gamma_{\theta,i}$

(g) find the probability distribution of θ_i, and fit it with a Gaussian function

(h) in the current time-window i, define L_i (the local value of L) and d_i (the local distance between the bead center and wall) as:

- case xy: $L_i = L$ (the global value defined in 6d), and $d_i = \sqrt{L^2 - \text{med}(r_i)^2}$, where med() indicates the median
- case xyz: $L_i = \sqrt{d_i^2 + \text{med}(r_i)^2}$, and $d_i = \text{med}(z_i)$, where med() indicates the median

(i) using SI eq.1, calculate the theoretical value of the drag $\gamma_{\theta_i,\text{th}}(d_i)$ for the given $r_i, \theta_i, L_i, s_{\text{min}}$

8. Calculate in each time-window the bending stiffness of the hook (of length $L_{\text{hook}} = 60$ nm) from the equipartition theorem, as:

(a) $EI_{sig} = \frac{k_B T}{\sigma_{\theta_i}^2} L_{\text{hook}}$, where the variance $\sigma_{\theta_i}^2$ is calculated directly from the signal θ_i

(b) $EI_{gaus} = \frac{k_B T}{\sigma_{\theta_i}^2} L_{\text{hook}}$, where $\sigma_{\theta_i}^2$ is variance of the Gaussian fit to the distribution of θ_i, found in 7g

(c) $EI_{lor} = 2\pi \gamma_\theta f_c L_{\text{hook}}$ where the drag γ_θ and the corner frequency f_c are obtained from Lorentzian fit of $PSD_{\theta_i}(f)$ found in 7f

These three methods to estimate of EI are not fully independent, and the difference between them is used as an internal consistency check. Only the value of EI_{lor} is kept in the following. This step 8 is relevant only for when the input s_{min} is the optimal value.

9. Calculate and return the value of $\text{MSE}(\gamma_\theta, \gamma_{\theta,\text{th}})$, the Mean Square Error between the experimental and theoretical drag (found in 7f and 7i, respectively), a function of the choice of s_{min}

As mentioned above, for each trace, the optimal value for the offset s_{min} is found by automatically minimizing (we use the python function `scipy.optimize.minimize`) the value of $\text{MSE}(\{\gamma_{\theta,i}\}, \{\gamma_{\theta,\text{th,i}}\})$, returned by the function `radial_analysis`$(x, y(, z), s_{\text{min}})$, by varying

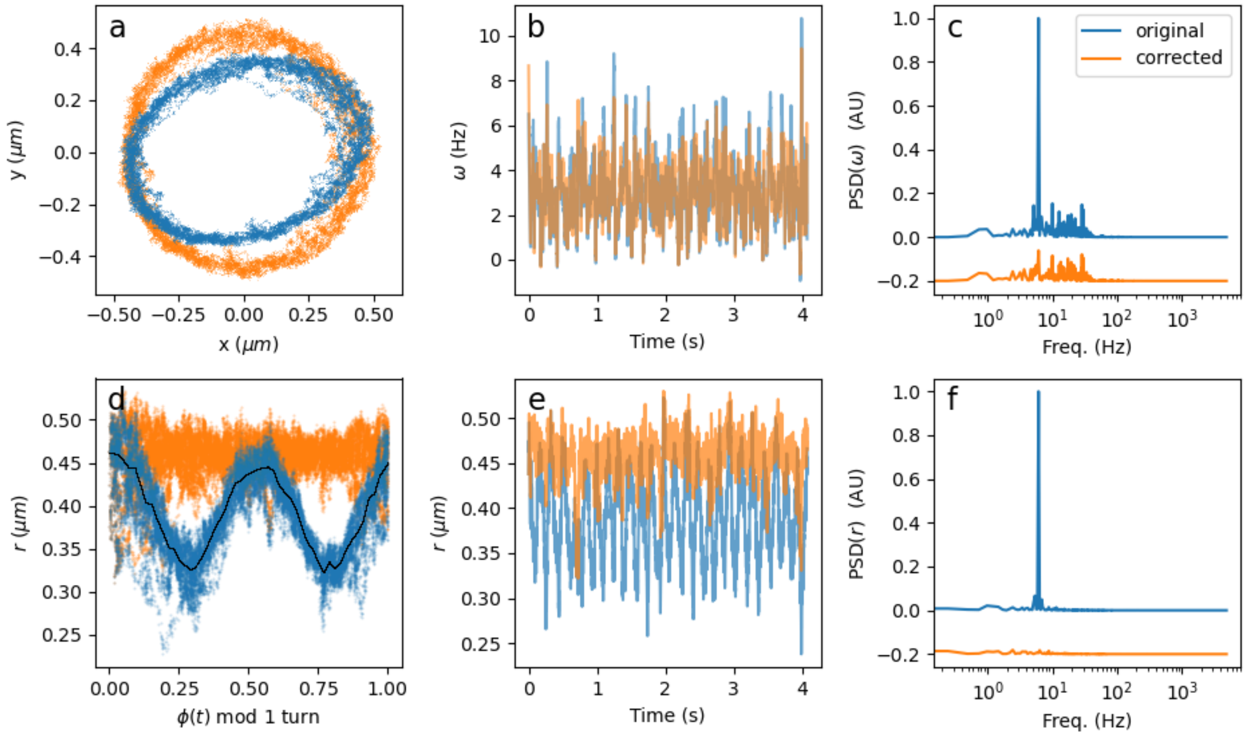

Supplementary Figure 7: Example of corrections performed on the experimental traces. In all panels, the original data are in blue, and the corrected data are in orange. In a-c the correction consists in fitting an ellipse on the trajectory and scale it to a circle. The spurious periodic modulation in the speed trace due to the ellipticity of the trajectory is in this way removed. In e-f the correction consists in removing directly from the signal (here the radius $r(t)$) the 1-turn periodic modulation. a) Portion of the x, y trajectory of a $R_b = 1000$ nm bead. b) Angular speed $\omega(t)$ of the bead, filtered by a running window median filter. c) Normalized spectrum of the speed $\omega(t)$, where the periodic modulation present in the original data (blue) is suppressed by the scaling of the trajectory (orange, vertically shifted for clarity). d) The original signal $r(t) = \sqrt{x^2 + y^2}$ (blue) is strongly modulated every turn due to the ellipticity of the trajectory. By subtraction of a high degree polynomial fit, or equivalently of an interpolated signal, (black line) the modulation is mitigated (orange). See section 2.2 (7-c). e) Time trace $r(t)$ with and without the correction shown in d. f) Normalized spectrum of the original (blue) and corrected radius $r(t)$ (orange, vertically shifted for clarity).

the input value s_{min} alone. For each trace, after the optimal s_{min} is determined, the value of s_i (the gap between the bead and the membrane) can be found in each time window i by $s_i = L_i^2 - r_i^2 - R_b$. This is subsequently used to calculate the tangential drag $\gamma_\phi(s)$ (SI eq.12) and the motor torque $\tau = \gamma_\phi \omega$.

2.3 Corrections of the bead trajectory

In bead assays, the measured trajectory of the bead is rarely perfectly circular. It is very common to observe an elliptical x, y trajectory, and this is usually explained by assuming the real 3D trajectory to be circular, but lying on a plane tilted with respect to the image plane, as can occur when the motor is on the side of the cell. The projection onto the image plane would then be observed as an ellipse. Observing bead trajectories in three dimensions, we have observed that the real 3D trajectory can also be not perfectly circular as assumed. This is probably the result of the interaction of the bead with the local topography of the cell.

Whenever the trajectory (either in 2D or 3D) differs from a circle, and if this perturbation is always present (as when it is due to the cell topography), the signals of interest obtained

from the trajectory (e.g $z(t)$, $\omega(t)$, $r(t)$) acquire a modulation that is periodic with respect to the position of the bead along the trajectory. Due to this periodicity, these signals can be corrected, as we show in one example in Fig.7.

In Fig.7a we show a bead that displays an elliptical x, y trajectory. We can perform two kinds of corrections on such traces:

1. (Fig.7a-c) We fit an ellipse to the x, y trajectory, and, using the ratio of the major to minor axis, we transform the x, y points into a circular trajectory. This alleviates the periodic modulation that otherwise affects the traces. In Fig.7a-c we show the effects of the correction on the speed trace $\omega(t)$ and its spectrum. In the time window shown, the angular speed $\omega(t)$ is strongly modulated at the frequency of rotation, as indicated by the peak of its spectrum. The modulation and the peak disappear after the correction.

2. We correct directly the signal affected by the periodic modulation (we use $r(t)$ in Fig.7d-f) by plotting it as a function of $mod(\phi(t)/2\pi, 1)$, effectively wrapping the signal onto itself every turn, Fig.7d. The 1-turn periodic modulation can be high pass filtered by fitting and subtracting a high order polynomial or an interpolated signal. In Fig.7d-f, we show the effect of this procedure on the signal $r(t)$ and its spectrum.

Supplementary Notes

1. Simulating a particle in a harmonic potential in presence of a drag gradient

A particle close to a rigid wall experiences an increasing drag as the distance to the wall decreases, as described by Faxen's and Brenner's equations (eq.11,10). Here, we explore by simulations the effect of this gradient on the diffusion of a particle trapped in a harmonic potential. The potential in our case is provided by the hook considered as an angular spring, providing a restoring force directed towards the equilibrium position. A similar potential could be provided by an optical trap. The analysis of the experimental data shows that, as motor speed and torque increase, the mean bead position moves towards the surface, the corner frequency f_c of the Lorentzian fit increases, the measured drag γ_θ increases, and the stiffness of the potential increases (Fig.3 of the main text). Here, we ask whether the increase in drag, induced by the wall proximity, can *alone* explain these observations, therefore excluding the mechanism of hook stiffening.

To answer this question, we have run Langevin simulations that reproduce our geometrical assumptions (we note that the choice of the algorithm is not trivial in the presence of viscosity gradients [4]). A bead of radius R_b can move along an arc of radius L. The bead position is described by the angle $\theta(t)$, and is trapped in a harmonic potential centered at an equilibrium angle θ_o. We can tailor the drag profile of the bead as a function of its position, and vary the equilibrium position in order to explore the behavior of limit cases. In Fig.8a-d, we allow the bead to fluctuate in the harmonic potential either i) in the absence of a wall, considering the constant bulk drag γ_o (in blue in all the panels), and ii) in the presence of the wall ($s = 50$ nm, red curves and points), where the drag dependency on θ is given by combining the components $\gamma_\perp$ and $\gamma_{||}$ both from Brenner and Faxen formulas (at a given θ, the drag $\gamma_\theta(\theta)$ is taken as the

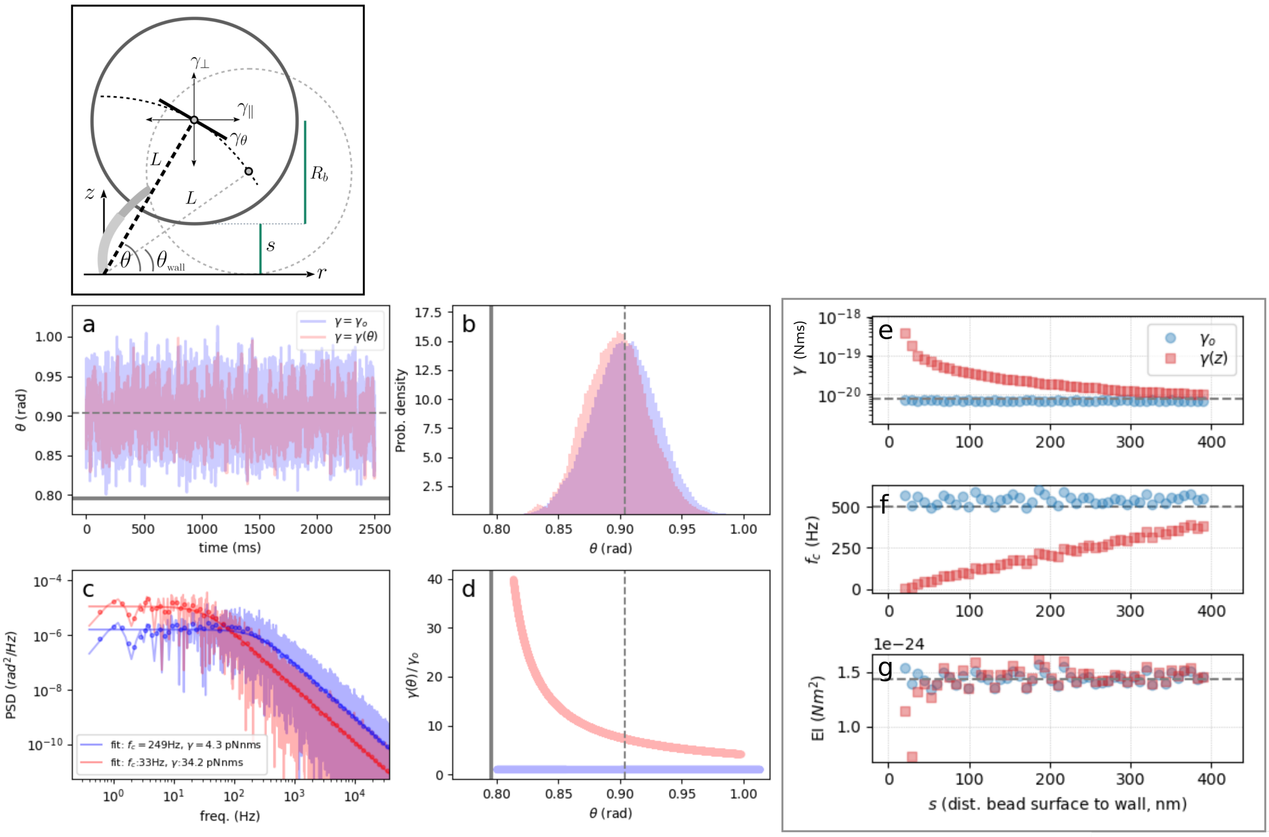

Supplementary Figure 8: Simulations of a particle in a harmonic potential with tailored drag dependency on position. **Top panel:** schematic geometry simulated, where the parameters have been chosen to be compatible with our measurements. A bead ($R_b = 500$ nm) can move on an arc of circle of radius $L = 700$ nm from the motor origin. The thermal fluctuations are simulated by a Langevin equation describing the evolution of the angle $\theta(t)$, which is assumed to be held at an equilibrium position by a harmonic potential centered at $\theta_o = 0.9$ rad ($s = 50$ nm, $r(\theta_o) = 430$ nm). The bead collides with an absorbing wall at $\theta = \theta_{\text{wall}}$. The harmonic potential has a stiffness $\kappa_\theta = 2\pi f_c \gamma_\theta$ defined by the corner frequency $f_c = 200$ Hz and a drag γ_θ which we set to either i) the constant bulk value $\gamma_o = 6\pi\eta R_b L^2 = 4.6$ pN nm s (blue curves and points) or ii) to the value of the function $\gamma_\theta(\theta)$ corrected by the presence of the wall by Brenner and Faxen theory (red curves and points). The noise term is delta-correlated. **a-d)** Simulation and analysis. a) Simulated traces of $\theta(t)$ for the two choices of the drag γ_θ. In the panels a,b,d the dashed line indicates the equilibrium angle θ_o, and the thick line indicates the position of the wall θ_{wall}. b) Probability distributions of the visited angle. c) Power spectral densities of θ. A Lorentzian fit (line) is performed on the equally log-spaced points shown. The parameters obtained from the fit are shown in the legend. In the case of $\gamma_\theta = \gamma_o$ (blue), they compare well with the input parameters of the simulation. The effect of the change in drag as a function of θ is reflected by the shift of the red spectrum with respect to the blue. d) Profiles of the drag $\gamma_\theta(\theta)/\gamma_o$ used in the simulation. The blue line is fixed at the value of 1, while the red increases in direction of the wall. **e,f,g)** Analysis results of simulations at varying distance s. The three panels show the values returned by the fit of the PSD of θ for the drag γ (panel e), the corner frequency (f_c, panel f), and the hook stiffness $EI = 2\pi f_c \gamma L_{\text{hook}}$ (panel g), in simulations (like those shown in panel a) where the distance s from the wall was changed, changing the equilibrium angle θ_o.

maximum between the expressions of Brenner and Faxen, see SI sec.2.1. The resulting drag used in the simulation as a function of the angle θ for the two cases is shown in Fig.8d.

We then treat the simulated θ displacement as an experimental trace. Its probability distribution, at the chosen distance $s = 50$ nm from the wall, is affected (0.01 rad) with respect to the simulation in the absence of the wall, as the particle spends more time in the region of higher effective viscosity. This occurs also in the experiment, but we note that the experimentally measured drag is only 4-5 times higher than bulk, while in the simulation a similar shift of the probability distribution is achieved with a drag 10-30 times higher than bulk. As in the experiment, in Fig.8c, we fit the PSD of the simulated $\theta(t)$ to a Lorentzian, obtaining a corner frequency f_c and an effective drag γ. In absence of the wall (blue spectrum), the fit returns the input parameters as expected, within the error. In proximity to the wall (red), despite the θ-dependency of the drag, the spectrum (red in Fig.8) maintains overall a Lorentzian shape. With respect to the case of constant drag, the spectrum is now shifted, giving rise to both a reduced corner frequency f_c, and an increased effective drag γ (see the legend of panel c). Due to the fact that the angular stiffness ($2\pi\gamma f_c L^2$) is proportional to both f_c and γ, this results in a stiffness that does not change significantly in presence of the wall. Moreover, while the increase of the fit drag goes in the direction of the experimental observation (although higher than in the experiment), a concomitant decrease of the corner frequency, and the resultant unaffected stiffness are not in agreement with our experimental observations. In panels e-g), we show the result of the PSD fit analysis performed while changing in the simulations the gap s between the bead and the wall. In presence of the wall and for a decreasing s, the fit drag increases (red points in panel e), the fit corner frequency decreases (panel f), and the stiffness remains at the same value as in absence of the wall. This is in disagreement with the experimental observations.

In conclusion, these simulations and their analysis show that the hydrodynamic effects due to a rigid wall, while they can account for qualitative features like the shift of the distribution towards higher viscosity and an increased drag obtained from the PSD, fail to explain the increase both in corner frequency and stiffness observed in the experiment. Therefore, the dynamic stiffening of the hook remains a valid mechanism to explain our data.

2. Bend-twist coupling, persistence length, and Young's modulus

In the classical continuum description of a rod, bending and twisting are independent. Following the treatment and the definitions used for DNA [10, 13], a coupling between the two can be inserted, writing the elastic free energy as,

$$F = \frac{1}{2} k_B T \int_0^L [A(\Omega_1^2 + \Omega_2^2) + C\Omega_3^2 + 2G\Omega_2\Omega_3]\, ds \tag{13}$$

Here, an orthonormal frame of three vectors $\{e_1, e_2, e_3\}$ is used to characterize each point of the rod, where e_3 is tangent to the curve. Three corresponding rotation vectors $\{\Omega_1, \Omega_2, \Omega_3\}$ (dimensions [rad/m]) connect adjacent local frames e_i, and describe any deformation of the relaxed configuration. Variations in e_1 and e_2 indicate bending along the two orthogonal directions, while variations in e_3 indicate twist. The parameter s is the arc-length of the curve, and L is the total length. The lengths A, C, and G denote the persistence lengths for bending, twist, and bend-twist coupling, respectively. $k_B T$ is the thermal energy. Assuming the simplest

EI (Nm^2)	Bacterial strain	Note	Ref.
$1.2 \pm 0.4 \cdot 10^{-25} \to 27 \pm 9 \cdot 10^{-25}$	*Escherichia coli*	Relaxed→Loaded	This study
$3.6 \pm 0.4 \cdot 10^{-26}$	*Vibrio alginolyticus*	Relaxed	[15]
$2.2 \pm 0.4 \cdot 10^{-25}$	*Vibrio alginolyticus*	Loaded	[15]
$1.6 \cdot 10^{-28}$	*Escherichia coli*		[14]
$3.0 \cdot 10^{-28}$	*Salmonella typhimurium*		[14]
$4.0 \cdot 10^{-28}$	*Vibrio cholerae*		[14]
$4.8 \cdot 10^{-28}$	*Vibrio parahaemolyticus*		[14]
$5 \cdot 10^{-28} - 5 \cdot 10^{-27}$	*Salmonella typhimurium*	Theoretical	[5]

Supplementary Table 1: Measurements of the bacterial hook bending stiffness EI.

case of constant quantities to resolve the integral, taking $\Omega_1 = 0$ and defining the twist and bending angle respectively as $\theta_T = \Omega_3 L$ and $\theta_B = \Omega_2 L$, the energy can be written as

$$F = \frac{k_B T}{2L}(A\theta_B^2 + C\theta_T^2 + 2G\theta_B\theta_T) \tag{14}$$

Twist and bend angle are confined in a parabolic potential well, with coupling. The restoring twist and bend torque can be written as

$$\tau_T = -\frac{\partial F}{\partial \theta_T} = -\frac{k_B T}{L}(C\theta_T + G\theta_B) \tag{15}$$

$$\tau_B = -\frac{\partial F}{\partial \theta_B} = -\frac{k_B T}{L}(A\theta_B + G\theta_T) \tag{16}$$

The presence of the coupling introduced in this manner adds an offset to the restoring torque. This is a term that, being not dependent on the variable, does not influence the stiffness (equal to $\partial\tau_i/\partial\theta_i$). Therefore, such coupling can shift the equilibrium angles, but cannot explain a change in stiffness.

In absence of coupling or for relaxed twist ($G\theta_T = 0$), one retrieves the linear relationship between restoring torque and angle, which for bending reads $\tau_B = \frac{k_B T A}{L}\theta_B = \frac{EI_o}{L}\theta_B$, where $EI_o = k_B T A$ is the bending stiffness of the relaxed hook ($\theta_T = 0$). As noted in the main text, our measurements are not performed on a perfectly twist-relaxed hook; but, for low torque (low load and low speed, $\tau < 100$ pN nm) our data ($EI_o = 1.2 \pm 0.4 \cdot 10^{-25}$ Nm^2) are compatible with the value found in torsionally relaxed hooks of *V. alginolyticus* ($EI_o = 3.6 \pm 0.4 \cdot 10^{-26}$ Nm^2 [15]). We note that the persistence length associated to this value of the bending stiffness is $A = EI_o/(k_B T) \sim 8\,\mu$m. As noted in [15], an EI_o two orders of magnitude lower has been measured by electron microscopy [14], yielding a persistence length of the order of the length of the hook. Our measurements and those described in [15], both based on the fluctuations of the hook in its native environment (and thus without the possible perturbations due to imaging in electron microscopy), indicate that the hook is probably not as soft as often pictured. However, we note that even with a persistence length of 8 μm, much longer than its dimensions, a stiffness $EI_o = 3.6 \pm 0.4 \cdot 10^{-26}$ Nm^2 allows thermal fluctuations of 10-20 degrees in *V. alginolyticus* [15]. In Table 1 we summarize the existing measurements of the hook bending stiffness.

Moreover, the area moment of inertia I (calculated for a hollow cylinder from the cross section as $I = \frac{\pi}{4}(R^4 - r^4)$, where R and r are the external and internal radii, respectively)

allows us to estimate the Young's modulus of the hook from the measured EI, which we find in the range $10^7 - 10^9$ Pa, depending on the choice of R and r (this simple result is for a homogeneous material, while the hook displays radial inhomogeneity [5]). This is higher, by one to two orders of magnitude, than theoretical predictions [5], which are dependent on the experimental value, and its uncertainty, of the hook shear modulus G [2].

3. Effect of torque fluctuations

We describe the fact that, in general, θ tends to decrease with increasing torque (or speed). Due to this relationship, fluctuations in motor torque (related to the stochastic dynamics of the motor) could contribute to the measured fluctuations in θ, therefore affecting the value of EI (proportional to $1/\sigma_\theta^2$, where σ_θ^2 is the variance of θ in a time window), the true value of which should rely on thermal fluctuations only. However, the plot of θ versus torque for all the traces (see SI Fig.9a, and SI Fig.3a) shows that overall, with a large variability among traces, the decrease in θ with torque stops at a torque of $\sim$ 500 pN nm. On the other hand, the plot of EI versus torque (see Fig.9b, same as Fig.5a of the main text) increases up to torques of $\sim$ 1000 pN nm. This difference in "cut-off torque" value between EI and θ as a function of torque suggests that the effect on EI, if any, would occur at torques below 500 pN nm. Moreover, both the absolute values of EI and its trend with increasing torque are in agreement with the results described by [15] (see SI Table 1). In particular, the value of EI of the relaxed hook was measured by Son et al. on motors where the speed was truly zero, observing fluctuations of pure thermal origin. In our work, instead, a small positive torque is required for the measurement. The two works, with different techniques, yield comparable values of EI for the relaxed state (in our case, at torques lower than 500 pN nm), in which the variation of θ with torque is the highest. This builds further confidence that our measurements are free from such an artifact, also at low torque. Finally, if speed fluctuations (or relative speed fluctuations) affect the value of EI, a trend should be apparent in the plot of EI versus σ_ω (or $\sigma_\omega/\omega = \sigma_\tau/\tau$) encompassing the three loads measured (where σ_ω is the standard deviation of the angular speed ω in a time-window, and τ is the torque). In SI Fig.9c,d such a clear relationship cannot be observed for the three loads in the plots of EI versus speed or relative speed. On the contrary, a clear relation among the three loads is only present in the plot of EI versus torque, which supports our conclusion.

4. Effect of centrifugal force

A simple calculation shows that the bending of the hook due to the centrifugal force during rotation is not sufficient to explain the observed movement of the bead towards the membrane. A bead of density ρ_b, radius R_b, and mass $m_b = \frac{4}{3}\pi R_b^3 \rho_b$, rotating with an angular speed ω on a circular trajectory of radius r, is responsible for a centripetal and centrifugal force $F = m_b \omega^2 r$. Considering the limiting situation where the hook is a vertical solid cantilever anchored at one extremity, its stiffness is $k_c = 3EI/L^3$, where L is the lever arm, and the maximum deflection under the action of a constant force is $\delta_B = \frac{FL^3}{3EI}$. Considering a latex bead ($\rho_b = 1$ g/cm^3) with $R_b = 0.5$ μm, $m_b = 5 \cdot 10^{-16}$ kg, rotating at $\omega = 2\pi \cdot 50$ rad/s, on a trajectory of radius $r = 200$ nm, the centrifugal force is of the order of $F \sim 10^{-17}$ N. Such force on a cantilever having the experimental value of the bending stiffness $EI = 10^{-25}$ Nm^2 would induce a maximum

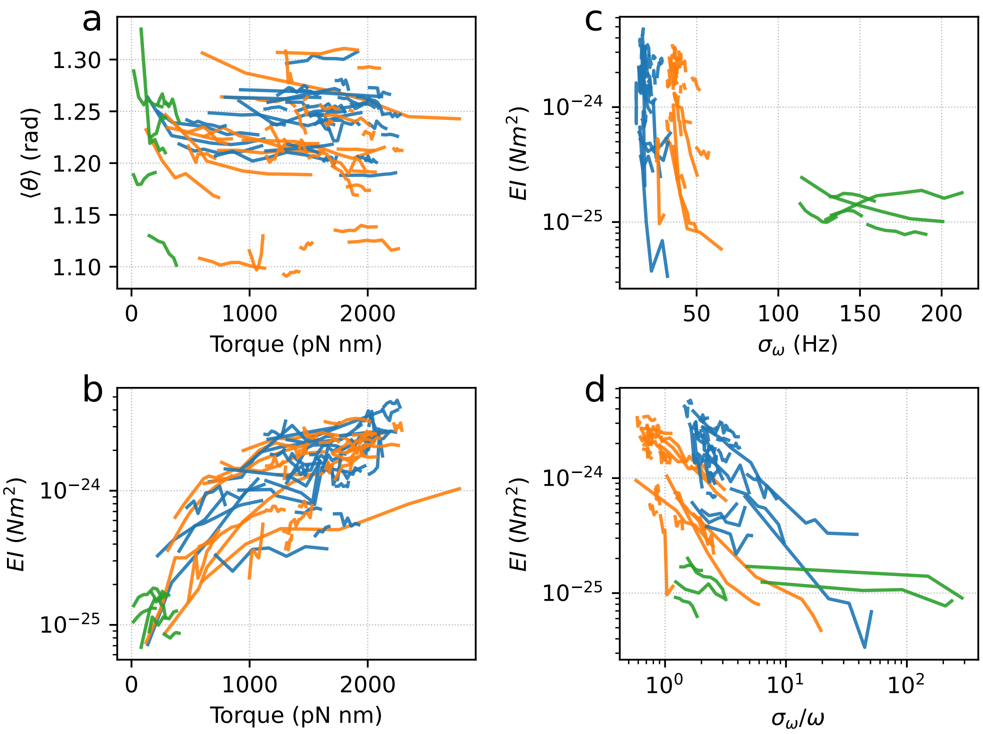

Supplementary Figure 9: a) Change of angle θ, averaged in each time-window of all the traces. b) Hook bending stiffness EI as a function of torque (same as Fig.5a of the main text). c) Hook bending stiffness EI as a function of angular speed standard deviation σ_ω. d) Hook bending stiffness EI as a function of relative speed standard deviation σ_ω/ω. As in the main text, the colors green, orange and blue correspond to beads of radius $R_b = 250, 500, 1000$ nm respectively.

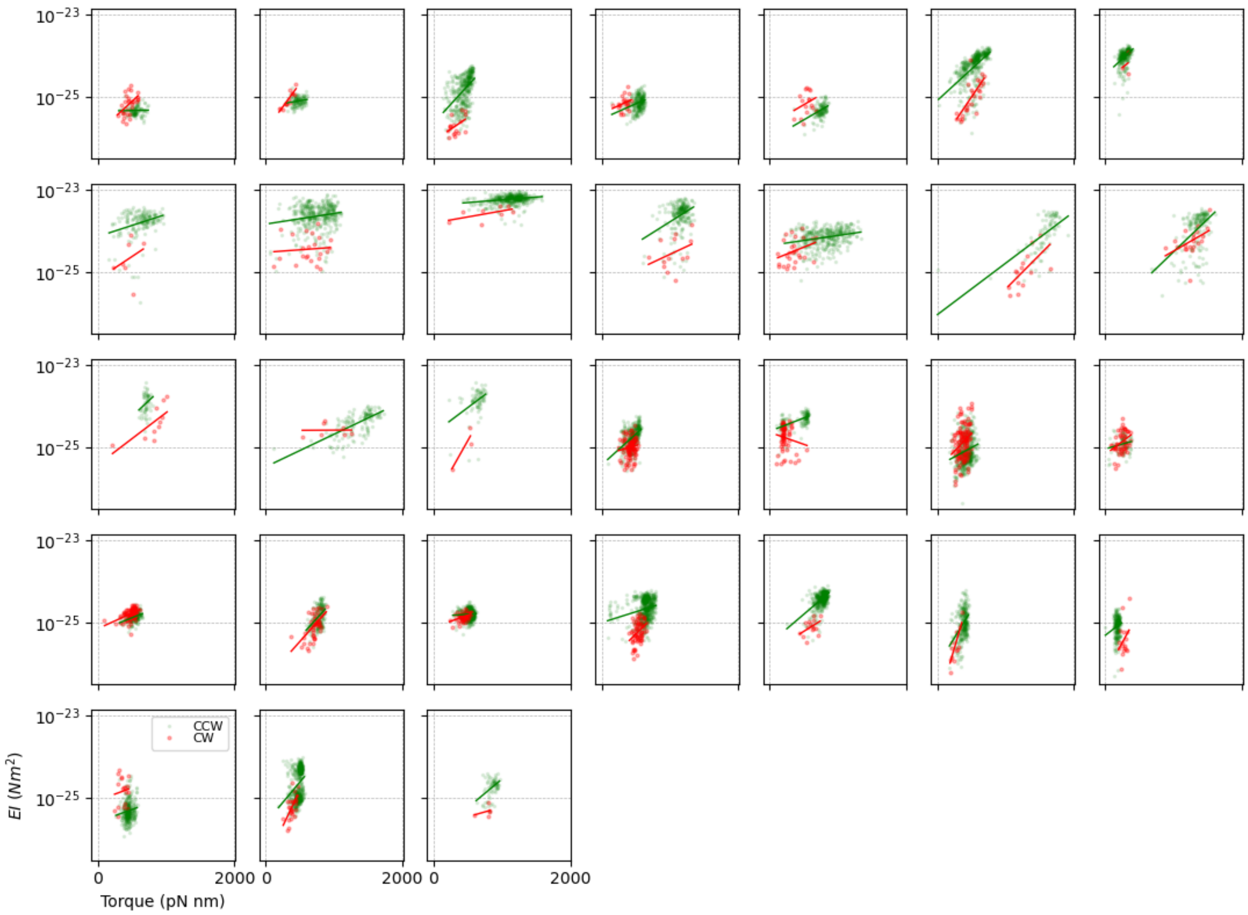

Supplementary Figure 10: Hook bending stiffness under opposite twists. The bending stiffness EI is measured from resurrection traces of a switching strain, separating the hook response when the motor rotates CCW and CW. Each panel corresponds to a different resurrection trace of different motors rotating beads with $R_b = 1000$ and 500 nm. Red: CW, green: CCW, linear fits are indicated by the lines.

deflection of only $\delta_B \sim 10^{-12}$ m (considering the bead radius as a lever arm).

5. Comparison of hook bending stiffening under opposite twists

Given the heterogeneity of the mechanical responses of different hooks (Fig.5 of the main text), we aimed at comparing the bending stiffness EI of a single hook dynamically subject to opposite twists during the resurrection of the motor. This can be done by analyzing resurrection traces where the motor switches between CCW and CW rotation. To probe the entire response of EI as a function of torque, we used large beads ($R_b = 500, 1000$nm). Using the *E. coli* parent strain where *cheY* is not deleted resulted in resurrection traces where the motors switched direction most frequently at large stator number and high torque, while at low stator number and low torque very few switches to CW were observed. This prevented the quantification of EI at low torque. With a Δ*cheRB* mutant we observed more frequent CW rotation intervals at low torque, and the results of these experiments are shown in Fig. 10.

The analysis of the signals described above, based on quantities extracted in time windows of a few seconds, works best when long dwell times with constant speed (either CCW or CW)

Name	Primer sequence (5' - 3')
tapfwd	TGCAGTTACAAATTGCGCCAGTGGTATCCTGAAGT
	GATTGAGAAGGCGCTCGCCTTACGCCCCGCCCTGC
cheYrev	AACCAAAAATTTAAGTTCTTTATCCGCCATTTCA
	CACTCCTGATTTAAATCTAGACTATATTACCCTGTT
cheRBfwd	GTCGCGTGTGGCGGTATTTACCC
cheRBrev	CCGCCTGCCTGCAACTTATTGAGAG

Supplementary Table 2: Oligonucleotide list.

are present in the trace. While this is possible in CCW rotation resurrections, we found that the dwell times in CW rotation were insufficiently long. This would produce artifacts, by mixing CW and CCW speed in single analysis time-windows. We therefore located all the CW dwell times, removed them from the trace and concatenated them at the end, such that all the CW and CCW dwell times were grouped together. To avoid artifacts from analysing zero-speed regions and from the speed transitions between CCW and CW (of tens of ms time duration), we further removed from the analysis all points where the absolute speed $|\omega|$ is below a threshold of 1 Hz. The resulting trace can be analyzed as before, as the total time spent in the CW rotation is sufficient for the time-windowed analysis.

These complications make the results shown in Fig.10 less conclusive than those obtained with the non-switching strain. However, we can distinguish in a majority of cases that the bending stiffness EI measured in CCW rotation (green points) is larger than in the CW rotation (red points). Yet, we sometimes see the inverse, or cases where the EI values are very similar. This qualitatively suggests that an asymmetry may be present in the way the hook stiffens when twisted in the two directions, but more data would be required to provide a clear and quantitative answer.

5.1 cheRB mutant preparation

The MT02 strain was used as the recipient for inactivation of the *cheRB* genes using $\lambda-$red mediated recombination method as described in [16]. The Δ*cheRB* mutant, deleted for the receptors methyltransferase and methylesterase, contains a higher concentration of CheY-P than wild-type cells, and it has been shown that they spend a larger percentage of time rotating CW than wild-type cells [9], enabling a measurement of EI versus torque on individual motors rotating in both directions. The *cheRB::cat* construct was designed to avoid any polar effect by reversely orienting the cat gene, so there are no effects on *cheYZ* expression. Briefly, the chloramphenicol resistance gene (*cat*) was amplified from the pWRG100 plasmid [1] using primers tapfwd and cheYrev, which imparted flanking homologous regions upstream and downstream of the *cheRB* genes. The MT02 strain was first transformed with the pKD46 plasmid encoding the Red recombinase and electroporated with the amplified PCR fragment. The chloramphenicol-resistant recombinant clones were purified twice on LB plates supplemented with chloramphenicol (25 mg/L) and characterized by PCR using primers cheRBfwd and cheRBrev. Phage P1 was used to transduce the mutation into the parental MT02 strain, yielding *cheRB::cat* MT02 strain. The oligonucleotides used in this study are described in Table 2.

Supplementary References

[1] K. Blank, M. Hensel, and R. G. Gerlach. Rapid and highly efficient method for scarless mutagenesis within the salmonella enterica chromosome. *PloS One*, 6(1):e15763, 2011.

[2] S. M. Block, D. F. Blair, and H. C. Berg. Compliance of bacterial polyhooks measured with optical tweezers. *Cytom. J. Int. Soc. Anal. Cytol.*, 12(6):492–496, 1991.

[3] H. Brenner. The slow motion of a sphere through a viscous fluid towards a plane surface. *Chem. Eng. Sci.*, 16(3-4):242–251, 1961.

[4] H. W. de Haan and G. W. Slater. Translocation of a polymer through a nanopore across a viscosity gradient. *Phys. Rev. E*, 87(4):042604, 2013.

[5] T. C. Flynn and J. Ma. Theoretical analysis of twist/bend ratio and mechanical moduli of bacterial flagellar hook and filament. *Biophys. J.*, 86(5):3204–3210, 2004.

[6] A. J. Goldman, R. G. Cox, and H. Brenner. Slow viscous motion of a sphere parallel to a plane wall—i motion through a quiescent fluid. *Chem. Eng. Sci.*, 22(4):637–651, 1967.

[7] S. Hell, G. Reiner, C. Cremer, and E. H. Stelzer. Aberrations in confocal fluorescence microscopy induced by mismatches in refractive index. *Journal of microscopy*, 169(3):391–405, 1993.

[8] J. Leach, H. Mushfique, S. Keen, R. Di Leonardo, G. Ruocco, J. Cooper, and M. Padgett. Comparison of Faxén's correction for a microsphere translating or rotating near a surface. *Phys. Rev. E*, 79(2):026301, 2009.

[9] P. Lele, B. Hosu, and H. Berg. Dynamics of mechanosensing in the bacterial flagellar motor. *Proc Natl Acad Sci U S A.*, 110:11839–11844, 2013.

[10] J. F. Marko and E. D. Siggia. Bending and twisting elasticity of DNA. *Macromolecules*, 27(4):981–988, 1994.

[11] K. Namba, I. Yamashita, and F. Vonderviszt. Structure of the core and central channel of bacterial flagella. *Nature*, 342(6250):648–654, 1989.

[12] K. C. Neuman and S. M. Block. Optical trapping. *Rev. Sci. Instrum.*, 75(9):2787–2809, 2004.

[13] S. K. Nomidis, E. Skoruppa, E. Carlon, and J. F. Marko. Twist-bend coupling and the statistical mechanics of the twistable wormlike-chain model of DNA: Perturbation theory and beyond. *Phys. Rev. E*, 99(3):032414, 2019.

[14] A. Sen, R. K. Nandy, and A. N. Ghosh. Elasticity of flagellar hooks. *Microscopy*, 53(3):305–309, 2004.

[15] K. Son, J. S. Guasto, and R. Stocker. Bacteria can exploit a flagellar buckling instability to change direction. *Nat. Phys.*, 9(8):494–498, 2013.

[16] B. Wanner and K. Datsenko. One-step inactivation of chromosomal genes in escherichia coli k-12 using pcr products. *P Natl Acad Sci Usa*, 97(12):6640–6645, 2000.
